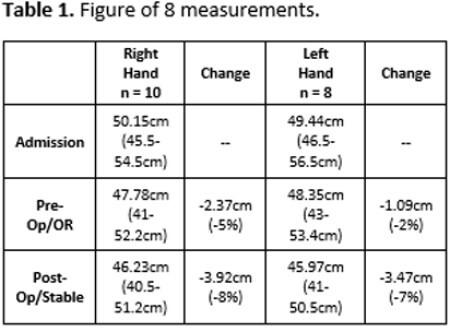# 767 Preoperative Edema Management for the Burned Hand

**DOI:** 10.1093/jbcr/irae036.309

**Published:** 2024-04-17

**Authors:** J Xavier Lucio, Walter R Anyan, Lily DuRose, Madelyn P Ellsworth, Irma D Fleming, Giavonni M Lewis, Callie M Thompson, Christopher R LaChapelle

**Affiliations:** University of Utah Health, Herriman, UT; University of Utah Health, Salt Lake City, UT; University of Utah Burn Center, Herriman, UT; University Of Utah Burn Critical Care, Salt Lake City, UT; University of Utah Health, Herriman, UT; University of Utah Health, Salt Lake City, UT; University of Utah Burn Center, Herriman, UT; University Of Utah Burn Critical Care, Salt Lake City, UT; University of Utah Health, Herriman, UT; University of Utah Health, Salt Lake City, UT; University of Utah Burn Center, Herriman, UT; University Of Utah Burn Critical Care, Salt Lake City, UT; University of Utah Health, Herriman, UT; University of Utah Health, Salt Lake City, UT; University of Utah Burn Center, Herriman, UT; University Of Utah Burn Critical Care, Salt Lake City, UT; University of Utah Health, Herriman, UT; University of Utah Health, Salt Lake City, UT; University of Utah Burn Center, Herriman, UT; University Of Utah Burn Critical Care, Salt Lake City, UT; University of Utah Health, Herriman, UT; University of Utah Health, Salt Lake City, UT; University of Utah Burn Center, Herriman, UT; University Of Utah Burn Critical Care, Salt Lake City, UT; University of Utah Health, Herriman, UT; University of Utah Health, Salt Lake City, UT; University of Utah Burn Center, Herriman, UT; University Of Utah Burn Critical Care, Salt Lake City, UT; University of Utah Health, Herriman, UT; University of Utah Health, Salt Lake City, UT; University of Utah Burn Center, Herriman, UT; University Of Utah Burn Critical Care, Salt Lake City, UT

## Abstract

**Introduction:**

At admission, we manage hand burns in four ways: (1) pre-operative hand edema control through elevation and application of ace bandages over the dorsal hand, (2) range of motion (ROM) protocols based on injury depth are used to restrict ROM to the digits to avoid injury to the ligamentous/tendinous structures of the hand/digit, (3) use of an intrinsic plus position hand splint, and (4) applying a silver sulfadiazine, gauze and netting dressing.

This practice has done an unsatisfactory job at reducing hand edema, resulted in prolonged immobilization and ROM restrictions, limited our patients’ ability to use their hands immediately post injury and ultimately lead to suboptimal functional outcomes at discharge.

As result, our center initiated a more aggressive pre-operative edema management plan which includes a preoperative self-adherent compression dressing (SACD) and a consistent practice for hand elevation.

**Methods:**

Upon admission, a thorough hand assessment is done. Edema management is started within 24 hours of admit. The hand injury is placed in clear antibiotic ointment and SACD. Elevation is managed with pillows stacked so the hand is consistently above the bedrail. Figure of 8 measurements are taken every other day when SACD is changed. This process continues until the edema has stabilized at which time a glove compression is applied. If edema increases after the glove compression is started, the patient returns to a SACD until the edema remains stable.

**Results:**

A total of 10 patients were treated with this protocol (8 B hands, 2 R hands, 18 hands total). All subjects were right hand dominant, nine males, and ages ranged from 17-74 years of age. TBSA burn ranged from 1-24% with (n=6) having TBSA < 10%. Split thickness autograft was placed on 16 hands (9R/7L). Figure of 8 values decreased during the pre- and post-operative phases of care (Table 1). No hand infections were noted. Composite flexion was improved at D/C: 12 hands (67%) able to make a full composite fist, 4 hands (22%) unable and 2 hands (11%) were not allowed to attempt due to ROM restriction per the surgeon (Figure 1).

**Conclusions:**

This approach better manages edema associated with all depths of hand burns and leads to better functional outcomes at D/C.

**Applicability of Research to Practice:**

We anticipate that this practice will allow us to move beyond our restrictive ROM protocols and reconsider pre-operative splinting practices. Which, in turn, will allow us to be able to progress patients' hand function more rapidly.